# Persistent Lymphatic Ascites After Liver Transplantation: Identification of the Underlying Mechanism of Ascites Permits Successful Percutaneous Treatment

**DOI:** 10.1007/s00270-025-04332-z

**Published:** 2026-01-23

**Authors:** Alexey Gurevich, Priscilla Stecher, Peter L. Abt, Maarouf A. Hoteit, Gregory J. Nadolski, Deborah Rabinowitz, Maxim Itkin

**Affiliations:** 1 Section of Interventional Radiology, Department of Radiology, University of Pennsylvania, 1 Silverstein, 3400 Spruce St, Philadelphia, PA 19104 USA; 2 Transplant Division, Department of Surgery, University of Pennsylvania, Philadelphia, PA USA; 3 Division of Gastroenterology and Hepatology, Department of Medicine, University of Pennsylvania, Philadelphia, PA USA; 4 Division of Interventional Radiology, Department of Medical Imaging, Nemours Children’s Hospital, Wilmington, DE USA

**Keywords:** Chylous ascites, Hepatic lymphorrhea, Liver transplantation

## Abstract

**Purpose:**

Approximately 1–7% of patients develop persistent lymphatic ascites after liver transplantation. This study describes the diagnosis and treatment of lymphatic ascites in patients post liver transplantation, refractory to conservative therapy.

**Materials and Methods:**

A review of the prospectively collected database was conducted to identify patients who received interventions for persistent lymphatic ascites following liver transplantation. Patient demographics, baseline characteristics, imaging findings, procedural details, and follow-up information were gathered.

**Results:**

Four adult patients after orthotopic liver transplantation with chylous ascites (CA) and 4 pediatric patients after split liver transplantation with hepatic lymphorrhea (HL) were included in this study. CA patients were characterized by elevated triglycerides (1010 mg/dL, 442–1769), and HL patients were characterized by low serum albumin ascites gradient (SAAG < 1.1) and low triglycerides. In 3/4 patients with CA, dynamic contrast MR lymphangiography and intranodal lymphangiography demonstrated obstruction of the central lymphatic system. The mesenteric lymphatics were then embolized with either *n*-BCA glue or lipiodol. One-fourth patients had stenosis of the portal vein anastomosis, which was balloon dilated using a transjugular approach. All 4 patients reached resolution of ascites at a median of 27 days. In 3/4 patients presenting with HL, liver lymphangiography demonstrated extravasation of the contrast. That was embolized with either glue or lipiodol. One-fourth patient demonstrated no extravasation but significant lymphangiectasia. In all patients, there was a resolution of ascites at a median of 14 days after intervention.

**Conclusion:**

Three mechanisms of post-transplantation lymphatic ascites were identified: portal venous hypertension due to iatrogenic obstruction; obstruction of central lymphatics resulting in congestion of the mesenteric lymphatic system and mesenteric lymphatic leak; and liver lymphorrhea. Identification of the mechanism of ascites allowed for successful percutaneous treatment in all patients.

**Graphical Abstract:**

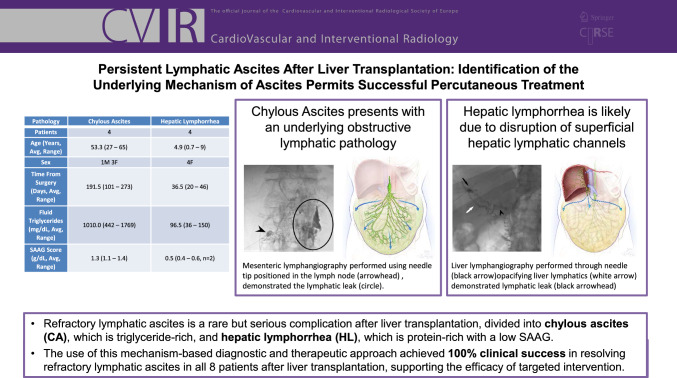

## Introduction

Persistent lymphatic origin ascites is a rare but serious complication following liver transplantation. There are two distinct types of lymphatic origin ascites: chylous ascites (CA) and hepatic lymphorrhea (HL), each characterized by specific fluid compositions and clinical implications.

Approximately 25–50% of the body's total lymph production originates from the liver, highlighting its importance in lymphatic circulation [1–3]. Most of the liver’s lymphatic vessels converge at the porta hepatis, ultimately draining into the thoracic duct (TD) [2, 4]. During liver transplantation, surgical manipulation, particularly extensive dissection around the porta hepatis, hepatoduodenal ligament, and retroperitoneum, can damage the lymphatic channels [5–8]. This disruption can result in HL post-transplantation characterized by the accumulation of high-protein (low serum albumin ascites gradient (SAAG)), low-triglyceride fluid in the abdomen originating from the new liver [7, 9]. Alternatively, CA originates from the lymphatic vessels in the intestines, leading to the accumulation of triglyceride-rich lymphatic fluid (chyle) in the peritoneal cavity [7, 10–13].

The reported incidence of lymphatic ascites after liver transplantation ranges from 1 to 4% in adults and 2–7% in pediatric cases [7, 9, 11]. Persistent ascites not amenable to medical therapy is even rarer, with the true incidence remaining unknown due to limited data and frequent underdiagnosis [8, 13]. Several risk factors have been identified for both conditions. These include the use of certain surgical techniques, such as the LigaSure vessel sealing system, prolonged operative time, pre-transplant ascites, and low albumin levels [7, 11].

Treatment of ascites starts with conservative measures like dietary modifications, paracentesis, and the administration of somatostatin analogs like octreotide [5, 7, 11, 12]. These interventions aim to reduce intestinal lymph production, thus promoting lymphatic healing. However, when conservative approaches prove ineffective, percutaneous interventions aimed at hepatic or mesenteric lymphatic systems have emerged as a promising alternative [8, 10]. These minimally invasive procedures are performed under image guidance, allowing for direct access to the hepatic and mesenteric lymphatic systems, enabling the targeted delivery of embolic agents to seal leaking vessels [2, 8, 10, 13]. In this study, we aim to stratify lymphatic ascites based on the origin of the leak and provide targeted solutions that align with the pathophysiology responsible for refractory lymphatic ascites after liver transplantation.

## Materials and Methods

### Patient Demographics and Study Design

This prospectively collected cohort study was approved by the local institutional review board under protocol # 834191. The patients were either adult recipients of a deceased donor orthotopic liver transplant (OLT) or pediatric recipients, with 3 patients receiving a living split liver transplant (SLT) and 1 patient receiving a deceased SLT. Patient demographics such as age, sex, fluid chemistry, length of the symptoms, imaging findings, clinical success of the intervention, time to resolution, and follow-up information were collected.

All patients underwent conservative and medical therapy (ranging from 97 to 217 days) before referral for lymphatic evaluation, which includes ascitic fluid analysis, dynamic contrast-enhanced MR lymphangiogram (DCMRL), intranodal lymphangiography, as well as mesenteric and liver lymphangiography. All imaging and interventions were performed by a single operator with over 20 years of experience. Chylous ascites was defined as ascitic fluid with a concentration of triglycerides higher than 200 mg/dL on a regular or low-fat diet, with concurrent SAAG score above 1.1. Hepatic lymphorrhea was defined as ascitic fluid with a concentration of triglycerides less than 200 mg/dL, with a concurrent SAAG score below 1.1. Clinical success was interpreted as the resolution of ascites and need for persistent paracentesis within two months of the final intervention.

### Lymphatic Imaging

#### Dynamic Contrast-Enhanced MR Lymphangiogram (DCMRL)

Imaging was performed as previously described [14]. In brief, after the patient’s groins were prepped and draped, access to bilateral inguinal lymph nodes was obtained under ultrasound guidance by inserting a 25-gauge needle (BD, Franklin Lakes, NJ) and 0.2 mL/kg of gadoterate meglumine (Dotarem, Guerbet Group, Princeton, NJ) divided into two doses was injected. Imaging was performed using high spatial and temporal resolution magnetic resonance angiography (Syngo Time-resolved angiography With Stochastic Trajectories [TWIST]) and navigator-gated 3D flash IR sequence.

#### Cone Beam CT Lymphangiography

In the interventional radiology suite, the lymph nodes were accessed with a Hook Needle (SureAx Medical, San Diego, CA) and water-soluble contrast was then injected to confirm efferent flow from the lymph node. Approximately 0.2 mL/kg of water-soluble iodinated contrast was then injected into lymph nodes, until contrast was identified in the area of cisterna chyli and cone beam CT was then performed.

#### Intranodal Lymphangiography

Intranodal lymphangiography was performed as described previously [15, 16]. Briefly, bilateral inguinal lymph nodes are visualized with ultrasound and accessed with a 24-G Hook Needle (SureAx Medical). Water-soluble iodinated contrast is injected to confirm the intranodal positioning. Subsequently, an oil-based contrast material (Lipiodol, Guerbet Group, Princeton, NJ) is slowly infused until opacification of the cisterna chyli, below the manufacturer’s recommended 0.25 mL/kg. The cisterna is then accessed transabdominally with a 22-G needle under fluoroscopic guidance, and a wire is advanced into the TD, after which the wire is exchanged for a 2.3-Fr microcatheter (Renegade STC, Boston Scientific, MA) for TD lymphangiography. TD cannulation was not performed routinely, avoided in cases where liver or mesenteric lymphangiography revealed the leak.

#### Liver Lymphangiography

Liver lymphangiography was performed as described previously [2]. A 24-G Hook needle (SureAx Medical) was positioned into the periportal space under ultrasound guidance, and water-soluble contrast was then injected to opacify liver lymphatic ducts, and the stabilizing wire of the Hook needle was deployed. When lymphatic flow was confirmed, an oil-based contrast material (lipiodol) was injected to achieve better opacification and detection of a leak.

#### Mesenteric Lymphangiography

Mesenteric lymphangiography was performed as described previously [3]. In brief, a 24-G Hook needle (SureAx Medical) was positioned into the mesenteric lymph nodes using US guidance. Water-soluble contrast was then injected to confirm opacification of lymphatic channels, followed by injection of an oil-based contrast agent (lipiodol).

### Lymphatic Interventions

#### Liver Lymphatic Embolization

Liver intervention was performed as described previously [2]. The embolization was performed using n-butyl cyanoacrylate (n-BCA) glue (TRUFILL®, Codman Neuro, Raynham, MA) mixed 1:2 with lipiodol, or with lipiodol alone, and administered through a Hook needle, which was positioned in liver lymphatics as described above.

#### Mesenteric Lymphatic Embolization

Mesenteric lymphatic intervention was performed as described previously [3]. In brief, the embolization using n-BCA glue mixed 1:2 with lipiodol, or with lipiodol alone, was performed through a Hook needle, which was positioned in mesenteric lymphatics as described above.

## Results

### Demographics

Patient demographics data, timing in relation to liver transplantation surgery, fluid triglyceride levels, and SAAG scores are reported in Table 1. Patients ranged in age from 27 to 65 years (mean of 53.3 years) for CA and 0.7–9 years (mean of 4.9 years) for HL after split liver transplantation. Fluid triglyceride levels were elevated for all CA patients and low for all HL patients. SAAG was low for all tested HL patients (*n* = 2) and elevated for all CA patients (*n* = 4).

**Table 1:** Patient demographics

Pathology	Chylous ascites	Hepatic lymphorrhea
Patients	4	4
Age (years, Avg, range)	53.3 (27–65)	4.9 (0.7–9)
Sex	1M 3F	4F
Time from surgery (days, Avg, range)	191.5 (101–273)	36.5 (20–46)
Fluid triglycerides (mg/dL, Avg, range)	1010.0 (442–1769)	96.5 (36–150)
SAAG score (g/dL, Avg, range)	1.3 (1.1–1.4)	0.5 (0.4–0.6, *n* = 2)

*SAAG* Serum albumin ascites gradient

### Lymphatic Imaging and Outcome of Interventions

Procedural management was targeted at suspected pathophysiology causing CA and HL. Clinical outcomes and imaging findings are summarized in Table 2.

**Table 2:** Imaging and outcome of interventions

Pt #	Imaging	Imaging findings	Intraprocedure findings	Procedures	Clinical success	Time to resolution (days)	Follow-up (months)
*Chylous ascites*							
1	DCMRL, CTAP	Patent TD, portal vein stenosis	Portal vein stenosis	PV plasty × 2	Yes	37	42
2	DCMRL	No central propagation	Obstructed TD, extravasation on mesenteric lymphangiography	Mesenteric and RP embolization with lipiodol	Yes	6	31
3	DCMRL	No central propagation	Obstructed TD, extravasation on mesenteric lymphangiography	Mesenteric embolization × 2	Yes	2	22
4	DCMRL	Nondiagnostic study	Obstructed TD, reflux in mesenteric lymphatics	Mesenteric embolization with lipiodol	Yes	37	23
*Hepatic lymphorrhea*							
5	DCMRL	Normal central lymphatic flow	Liver lymphatic dilation, no leak	Liver lymphatic embolization with lipiodol × 2	Yes	42	57
6	DCMRL	Normal central lymphatic flow	Liver lymphatic leak	Liver lymphatic embolization	Yes	5	55
7	CBCTL	Obstructed TD	Liver lymphatic leak	Liver lymphatic embolization × 2	Yes	5	41
8	No	N/A	Liver lymphatic leak	Liver lymphatic embolization	YES	4	15

*DCMRL* dynamic contrast-enhanced MR lymphangiogram, *CTAP* CT abdomen and pelvis, *TD* thoracic duct, *CBCTL* cone beam CT lymphangiogram, *PV* plasty = portal vein angioplasty, *RP* retroperitoneal

#### Chylous Ascites

##### Portal Venous Obstruction

Patient 1 presented with CA had a portal vein anastomotic stenosis on cross-sectional imaging. The frequency of paracentesis was reduced after transhepatic portal vein angioplasty to 16 mm, and the reduction of gradient was from 7 to 1 mmHg. The need for paracentesis completely ceased with the subsequent repeat angioplasty to 18 mm 10 weeks later, with the repeat gradient decrease from 2 to 1 mmHg.

##### Central Lymphatic Obstruction

**Fig. 1:**
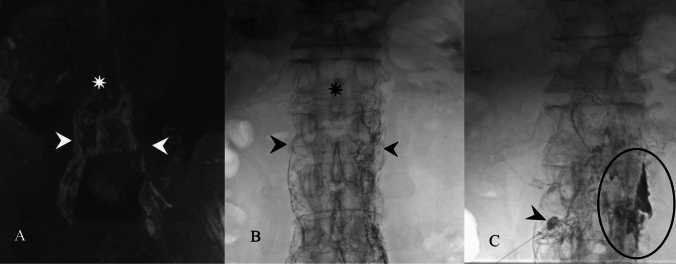
Patient 3: Dynamic contrast-enhanced MR lymphangiogram (**A**) and intranodal lymphangiography (**B**) demonstrating opacification of the retroperitoneal lymphatics (arrowheads) and no propagation of the contrast in the thoracic duct (star). Mesenteric lymphangiography (**C**), performed using the needle tip positioned in the lymph node, demonstrated the lymphatic leak (circle)

Patients 2, 3, and 4 presented with central lymphatic obstruction, demonstrated by poor propagation of contrast through the thoracic duct as seen on DCMRL (Fig. 1).

In patient 2, TD was absent on intranodal lymphangiography. Mesenteric lymphangiography demonstrated multiple mesenteric lymph nodes with no obvious mesenteric lymphatic propagation and a large retroperitoneal lymphatic mass, which was filled with lipiodol with subsequent resolution of CA.

**Fig. 2:**
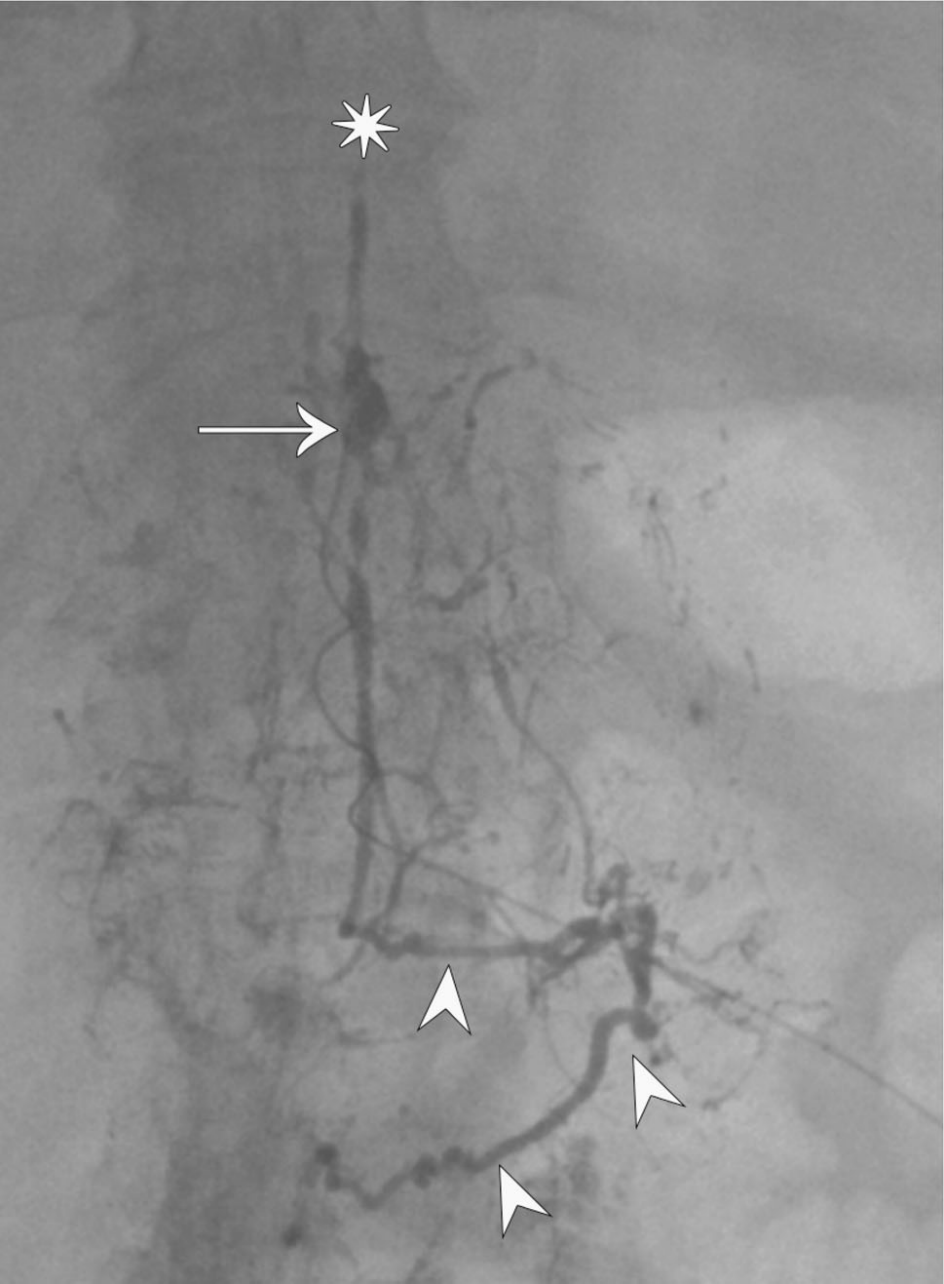
Patient 4: Contrast injected in the stump of the thoracic duct through a microcatheter inserted through cisterna chyli (arrow), demonstrates the occlusion of the thoracic duct (star) and reflux of the contrast in the mesenteric lymphatics (arrowheads)

In patient 3, TD was absent on intranodal lymphangiography. Mesenteric lymphangiography demonstrated an intraperitoneal leak, which was filled with lipiodol (Fig. 2A). Repeat mesenteric lymphangiography one week later did not redemonstrate the leak, and the patient’s CA subsequently resolved.

Patient 4 lymphangiogram revealed distal occlusion of TD and extensive mesenteric reflux. Mesenteric lymphangiography was performed with lipiodol, leading to resolution of CA (Fig. 2).

All patients remain ascites-free at an average of 29.5 ± 9.2 months post-intervention (ranging from 22 to 42 months).

#### Hepatic Lymphorrhea

In patient 5, DCMRL demonstrated patent TD. Liver lymphangiography demonstrated multiple dilated hepatic lymphatic vessels without an obvious leak, which were filled with lipiodol. Patient gradually improved with complete resolution of HL.

In patient 6, DCMRL demonstrated patent TD. Patient 7 had an obstruction of TD on cone beam CTL. Patient 8 did not have preprocedural imaging.

**Fig. 3:**
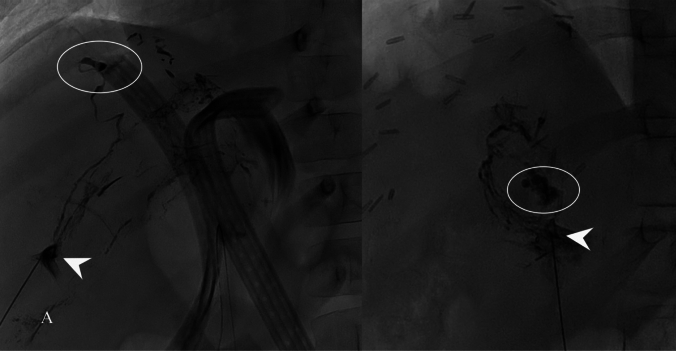
**A** Patient 6 and 8, liver lymphangiography performed through Hook Needle (SureAx Medical, San Diego, CA) positioned in the liver lymphatics (arrowhead) demonstrated lymphatic leak (circle)

Patients 6 through 8 underwent liver lymphangiography that demonstrated extravasation of contrast (Fig. 3). Embolization with a mixture of n-BCA and lipiodol (1:2) was performed with resolution of HL in all 3 patients. All HL patients remain ascites-free at an average of 42.0 ± 19.4 months post-intervention (ranging from 15 to 57 months).

## Discussion

This case series demonstrates three pathophysiological mechanisms of ascites in patients post liver transplantation. We used a combination of fluid analysis as well as pre- and intraprocedural imaging to differentiate between the three mechanisms and apply targeted percutaneous interventions. Recognizing these distinct mechanisms is crucial for effective management as they require tailored interventions aimed at addressing the specific causes of lymphatic leakage.

Persistent ascites (lasting longer than 4 weeks after transplant) occurs in approximately 5–10% of liver transplant recipients, the majority caused by peritoneal infection [17, 18]. Chylous ascites post liver transplantation is a rare complication, easily discernable due to its milky appearance and high triglyceride content [7]. In comparison, hepatic lymphorrhea represents a less well-recognized lymphatic origin complication due to low triglyceride fluid levels and high albumin, commonly mixed in with other etiologies of low SAAG, high protein ascites, and posing a diagnostic challenge.

The distinction between chylous ascites and hepatic lymphorrhea reflects fundamental differences in the chemical composition of lymphatic fluid depending on its anatomical source. Intestinal lymph, or chyle, is lipid-rich due to its role in nutrient absorption. In contrast, hepatic lymph is typically lipid-poor and protein-rich, with higher albumin content than chyle, reflecting the mechanism of liver lymph production through filtration of plasma from the sinusoid into the space of Disse, through very porous sinusoidal capillaries [19]. These compositional differences parallel the principles underlying SAAG, with low SAAG demonstrating a high concentration of albumin in ascitic fluid [20]. Appreciating these compositional nuances is essential for understanding the pathophysiology of post-transplant lymphatic ascites and for guiding accurate diagnostic and therapeutic strategies.

After the origin of ascites is established, the role of pre- and intra-procedure imaging is to confirm the diagnosis and establish the therapeutic approach. First, the cross-sectional imaging of the hepatic venous circulation is important to exclude either hepatic or portal vein obstruction and mesenteric venous congestion. If discovered, decompression of the portal vein and/or hepatic vein stenosis is performed as described in patient 1. Serial dilations of the portal vein anastomosis and improved mesenteric venous outflow successfully resolved chylous ascites.

Dynamic contrast-enhanced MR lymphangiography and cone beam CT lymphangiography were utilized for the evaluation of the TD, assisting in evaluating the level of potential obstruction within the central lymphatic system. Although minor mesenteric lymphatic disruptions from transplantation surgery typically do not lead to severe issues, in patients with pre-existing central lymphatic occlusion and elevated lymphatic pressure, this could act as a predisposing medical condition and a possible risk factor. In cases of TD obliteration, the only percutaneous treatment option is to perform mesenteric lymphangiography with oil-based iodinated contrast (lipiodol). The ability of the lipiodol to close the lymphatic leaks has been well described [3, 21–23]. The assumption is that the thick oily contrast blocks the small leaking lymphatic vessels with eventual sclerosis of the lymphatic microvasculature. This was successfully performed in half of the patients presented in this study.

Liver lymphorrhea is an underappreciated entity of ascites, previously highlighted primarily in Japanese literature. Guez et al. have described the embolization of liver lymphatics in a patient with liver lymphorrhea post Whipple procedure [10]. Naturally, hepatic lymphatics are transected in all patients undergoing liver transplantation, yet the complication remains rare, likely due to liver congestion and poor venous drainage. All 4 patients with HL in this study were children undergoing split liver transplantation. This type of liver transplantation requires division of the liver and imposes a high risk of damage to the complex hepatic lymphatic system, which includes both deep vessels originating in the periportal spaces and superficial vessels draining the liver capsule [4]. Three of the patients displayed frank extravasation and were successfully treated with n-BCA glue embolization, while the 4th patient displayed multiple lymphatic channels which were also successfully treated with lipiodol infusion alone.

The study’s limitations are evident in its relatively small sample size and the specialized nature of the patient population, which may restrict the broader applicability of the findings. Moreover, the retrospective analysis introduces inherent biases, potentially skewing the results and introducing possible lapses in data acquisition. Future prospective studies would be invaluable in validating these findings and refining treatment protocols, keeping in mind that rare diseases should always favor intention to treat.

## Conclusion

The successful management of refractory lymphatic ascites after liver transplantation hinges on accurately determining the specific underlying pathophysiological mechanism through a combination of fluid analysis and pre- and intraprocedural imaging. By classifying lymphatic ascites into three distinct mechanisms—portal venous hypertension due to iatrogenic obstruction, central lymphatic obstruction resulting in a mesenteric lymphatic leak, and hepatic lymphorrhea—this study facilitated a targeted interventional approach. Utilizing specific percutaneous treatments, such as portal vein angioplasty, mesenteric lymphatic embolization, and transhepatic lymphatic embolization, we achieved 100% clinical success in all eight patients with refractory lymphatic ascites, with resolution occurring at a mean of 17 days (ranging from 2 to 42) after intervention. The detailed diagnostic and therapeutic protocols presented here, which link mechanistic identification to tailored intervention, offer a highly reproducible strategy for managing this rare complication and support the scalability of this minimally invasive approach across other specialized centers.
